# The Correlation Between Tuberous Sclerosis Complex Genotype and Renal Angiomyolipoma Phenotype

**DOI:** 10.3389/fgene.2020.575750

**Published:** 2021-02-19

**Authors:** Nianyi Zhang, Xiaofang Wang, Zengqi Tang, Xiaonan Qiu, Zhixuan Guo, Danqi Huang, Hui Xiong, Qing Guo

**Affiliations:** ^1^Department of Dermatology, Sun Yat-sen Memorial Hospital, Sun Yat-sen University, Guangzhou, China; ^2^Department of Dermatology and Venerology, University of Chinese Academy of Sciences Shenzhen Hospital, Shenzhen, China; ^3^Guangdong Provincial Key Laboratory of Malignnt Tumor Epigenetic and Gene Regulation, Sun Yat-sen Memorial Hospital, Sun Yat-sen University, Guangzhou, China

**Keywords:** tuberous sclerosis complex, renal angiomyolipoma, genotype, phenotype, correlation assay

## Abstract

Tuberous sclerosis complex (TSC) is a rare multisystem autosomal dominant genetic disease that occurs between 1 in 6,000 and 1 in 10,000 live births. Additionally, renal angiomyolipoma is the most common form of renal disease in patients affected by TSC. Although a genetic mutation analysis of TSC is not rare, the correlation between the TSC gene mutation and renal angiomyolipoma phenotype is poorly understood. This study aims to analyze the mutation sites in 261 types of selected TSC patients. The results reveal that: (1) female patients develop more renal angiomyolipoma than male patients [*p* = 0.008, OR = 2.474, 95%CI (1.258–4.864)]; (2). The missense mutation of *TSC1* led to a higher risk of renal angiomyolipoma [*p* < 0.01, OR = 15, 95%CI (2.859–78.691)], and in contrast, showed a reduced risk in patients with frameshift mutation [*p* = 0.03, OR = 0.252, 95%CI (0.07–0.912)]; (3). Patients with *TSC2* mutations in the transcription activation domain 1 coding genes, had increased renal angiomyolipoma [*p* = 0.019, OR = 3.519, 95%CI (1.226–10.101)]. Therefore, our genotype-phenotype correlation study might shed light on the early monitoring and evaluation of renal angiomyolipoma in TSC patients.

## Introduction

Tuberous sclerosis complex (TSC), also known as Bourneville’s disease, is a rare autosomal dominant neurocutaneous syndrome. The estimated incidence rate of TSC is between of 1/6000 and 1/10,000 in live births and 1 in 20,000 in the population (Northrup et al., 2013). TSC is a genetic disorder that is characterized by the growth of benign hamartomas in various organs and is caused by *TSC1* or *TSC2* gene mutations (Ni et al., 2019). The *TSC1* gene is located on chromosome 9q34 and consists of 23 exons that encode the hamartin (Slegtenhorst et al., 1997) and the *TSC2* gene is located on chromosome 16p13 and consists of 42 exons that encode the tuberin (European Chromosome 16 Tuberous Sclerosis Consortium,, 1993). Interestingly, hamartin and tuberin form the TSC protein complex with TBC1 domain family member 7 (TBC1D7) (Dibble et al., 2012). Functionally, *TSC1* is necessary to maintain the stability, activity, and the correct intracellular localization of the TSC1-TSC2 complex (Rosner et al., 2008). *TSC2* contains a highly conserved GTPase activating protein that hydrolyzes the small GTPase Ras-homolog enriched in brain (RHEB), which inhibits the activation of mammalian rapamycin target protein 1 (mTOR1) which is vital in regulating cell growth and metabolism (Li et al., 2004). Thus, the *TSC1/2* gene mutations inactivate the TSC protein complex and aberrations of mTORC1 results in the occurrence of TSC.

Severe renal manifestations occur with a high frequency in patients carrying TSC mutations. The estimated incidence rates of renal manifestations, including renal angiomyolipoma (RAML), renal cysts, and renal cell carcinoma, in patients carrying TSC mutations range from 48 to 80%. Among these, RAML, which is a benign tumor, is the most common type, with an incidence rate of 34–80% (Rakowski et al., 2006). Of note, compared with sporadic RAML, TSC-RAML had an early onset, with bilateral and multiple lesions and faster growth (Rabenou and Charles, 2015). Although most of the RAML patients had no obvious clinical symptoms, a great proportion of patients experience abdominal pain, low blood volume shock, renal insufficiency, or end-stage renal disease which can even lead to mortality (Swärd et al., 2020; Vabret et al., 2020). Overall, RAML is considered to be the major cause of death in TSC patients (Amin et al., 2017).

Accumulating studies have suggested a correlation between the severity of clinical symptoms and *TSC1*, and *TSC2* gene mutations in patients with TSC (Lewis et al., 2004; Au et al., 2007; Bai et al., 2017). Nevertheless, the correlation between the types/sites of gene mutations and symptoms are fragmentary and therefore should be elucidated further (van Eeghen et al., 2012). The correlation between the genotype of the TSC gene and the RAML phenotype would benefit early detection and intervention as well as the prognosis and quality of life of patients with RAML.

## Materials and Methods

### Literature Search

An extensive and comprehensive literature search was conducted with regard to the relationship between TSC genotypes and RAML phenotypes using PubMed and Google. The search terms included “(RAML OR renal angiomyolipoma OR renal hamartoma) AND (TSC OR tuberous sclerosis complex OR Bourneville’s disease) AND (genotype OR mutation),”“(angiomyolipoma OR renal vascular smooth muscle lipoma OR renal tumors) AND (TSC OR tuberous sclerosis complex OR Bourneville’s disease) AND (genotype OR mutation),”“(TSC OR tuberous sclerosis complex OR Bourneville’s disease) AND (RAML OR renal angiomyolipoma OR renal hamartoma),” and “(TSC OR tuberous sclerosis complex OR Bourneville’s disease) AND (angiomyolipoma OR renal vascular smooth muscle lipoma OR renal tumor),” (TSC OR tuberous sclerosis complex OR Bourneville’s disease) AND (genotype OR phenotype). All retrieved papers were read to evaluate whether the reported patients met the inclusion criteria of the present study.

### Study Subjects

#### Inclusion Criteria

Patients (1) who met the 2012 NIH Tuberous Sclerosis Complex Diagnostic Criteria Updated (Northrup et al., 2013); (2) who underwent abdominal imaging examination (ultrasound, computed tomography or magnetic resonance imaging) for a definitive diagnosis of RAML; and (3) should undergo TSC gene testing to determine the pathogenic germline mutations.

#### Exclusion Criteria

Patients (1) who lacked abdominal imaging examination or did not undergo abdominal imaging examination for confirming the diagnosis of RAML; (2) with incorrect gene mutation information or if the base of the DNA mutation failed to comply the corresponding base in the reference sequence; (3) with two or more TSC gene variants detected, and pathogenic mutations could not be successfully determined; and (4) with only a specific mutation type or mutation site.

Based on the strict inclusion and exclusion criteria, 261 patients were enrolled in our study, of which 54 patients had the *TSC1* gene mutation and 207 patients had the *TSC2* gene mutation. The original data was collected from papers that were published from 1998 to April 2020. Patients were mainly recruited from six countries and regions including China, United States, Japan, Taiwan, Russia and Greece.

### Mutation Analysis

The TSC gene mutation information was verified and analyzed for each patient using ^1^Mutalyzer with the transcript reference number *TSC1* (NM_000368.4) or *TSC2* (NM_000548.3). The LOVD database was used to explore the exon number of ^2^*TSC1* and ^3^*TSC2*.

*TSC1* contains 23 exons, while exons 1 and 2 have no coding function (Avgeris et al., 2017). In addition, *TSC1* is classified as the N-terminal and C-terminal by exon 15 (van Eeghen et al., 2012). Hamartin has a potential transmembrane domain that is encoded by exon 6 (TMD, 127–144 amino acids) and a coiled-helix structural coiled coil domain that is encoded by exon 18–22 (CCD, 730–996 amino acids) (Ali et al., 2005). Of interest, hamartin also contains a tuberin interaction domain (TID) that is encoded by exons 8–11 (van Eeghen et al., 2012).

The *TSC2* gene contains 41 coding exons and 1 non-coding guiding exon (Satake et al., 1998). In previous studies, the number of exons were in disunity. In our study, we described all the TSC2 mutation sites according to the LOVD Databases with an encoding exon. Tuberin contains seven functional domains, that are arranged from the N-terminal to the C-terminal, and are as follows: Leucine zipper domain encoded by exon 3 (LZD, 81–98 amino acids), coiled coil domain 1 encoded by exon 10 (CCD1, 346–371 amino acids), coiled coil domain 2 encoded by exon 26 (CCD2, 1008–1021 amino acids), and transcription activation domain 1 encoded by exons 29–30 (TAD1, 1163–1259 amino acid), GTPase activating protein domain encoded by exons 34–38 (GAPD, 1517–1674 amino acids), transcription activation domain 2 encoded by exons 39–40 (TAD2, 1690–1744 amino acids) and the calmodulin-binding domain encoded by exons 40–41 (CaBD, amino acids 1740–1755) (Li et al., 2018). Due to overlapping amino acids between the TAD2 and CaBD domains, patients with partial amino acid changes that are located in the overlapping regions could not be located, and so two domains were included in one group during the analysis. Additionally, tuberin also has a hamartin interaction domain (HID). According to the location of encoding exon (E) and effect of the *TSC2* gene mutations, the affected amino acids were divided into three subsets, HID-TID (E1-E22), GAP domain (E34-E41), and mutations between the two regions (E23-E33) (van Eeghen et al., 2012).

Moreover, the mutation types were divided into a protein truncation mutation (PT) and a protein non-truncation (NT) mutation based on differential regulations of the gene mutation on the protein structure. PT comprises frameshift mutations, splice site mutations, nonsense mutations, and large fragment mutations. NT contains missense mutations and in-frame mutations (van Eeghen et al., 2012). Large fragment mutations were excluded from the study when the correlation between the functional domain gene mutation and RAML was present.

### Statistical Analysis

SPSS version 25.0 (IBM Corporation, Chicago, IL, United States) and GraphPad Prism version 8.0.2 were used for data analysis and illustration. A Chi-square test was used for categorical variables. When the expected value on contingency tables was below 5, then the results of the total sample size with ≥40 were corrected for continuity. When any expected value in the contingency table was below one or the total sample number was less than 40, then Fisher’s exact test was used. Binary logistic regression was used to evaluate the risk of developing RAML, and *p*-values of <0.05 were considered to be statistically significant.

## Results

### Patient Characteristics

**FIGURE 1:**
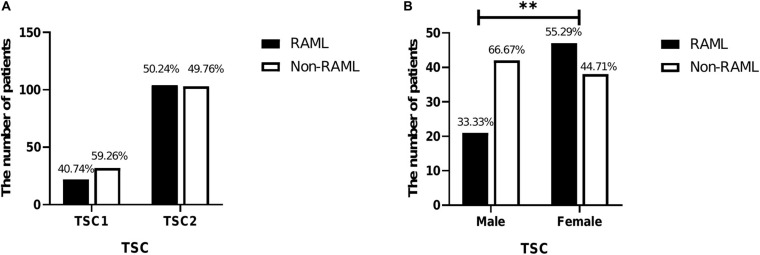
Characteristics of 261 patients included in this study. **(A)** Comparison of *TSC1* and *TSC2* mutations in RAML and non-RAML patients. **(B)** Comparison of genders in RAML and non-RAML of TSC patients; ^∗∗^*p* < 0.01.

A total of 261 patients were analyzed in this study. Briefly, 54 patients had *TSC1* mutations, and 22 of 54 (40.74%) had RAML. The remaining 207 patients had the *TSC2* mutation and 104 of 207 had RAML (50.24%). Although a higher rate of RAML was observed in patients with *TSC2* gene mutations than those with the *TSC1* gene mutation, there was no statistical significance [*p* = 0.213, OR = 0.681, 95%CI (0.371–1.25)] (Figure 1A). Of all the 261 patients, the incidence rate of RAML was shown to be 48.28%. Of the 148 patients knowns genders, 63 were male (42.57%) and 85 were female (57.43%). The incidence of RAML was higher in females (47 out of 85, 55.29%) than in males (21 out of 63, 33.33%), [*p* = 0.008, OR = 2.474, 95%CI (1.258–4.864)] (Figure 1B), indicating that female TSC patients might be more susceptible to RAML.

### Correlation Between *TSC1* Mutations and RAML

**FIGURE 2:**
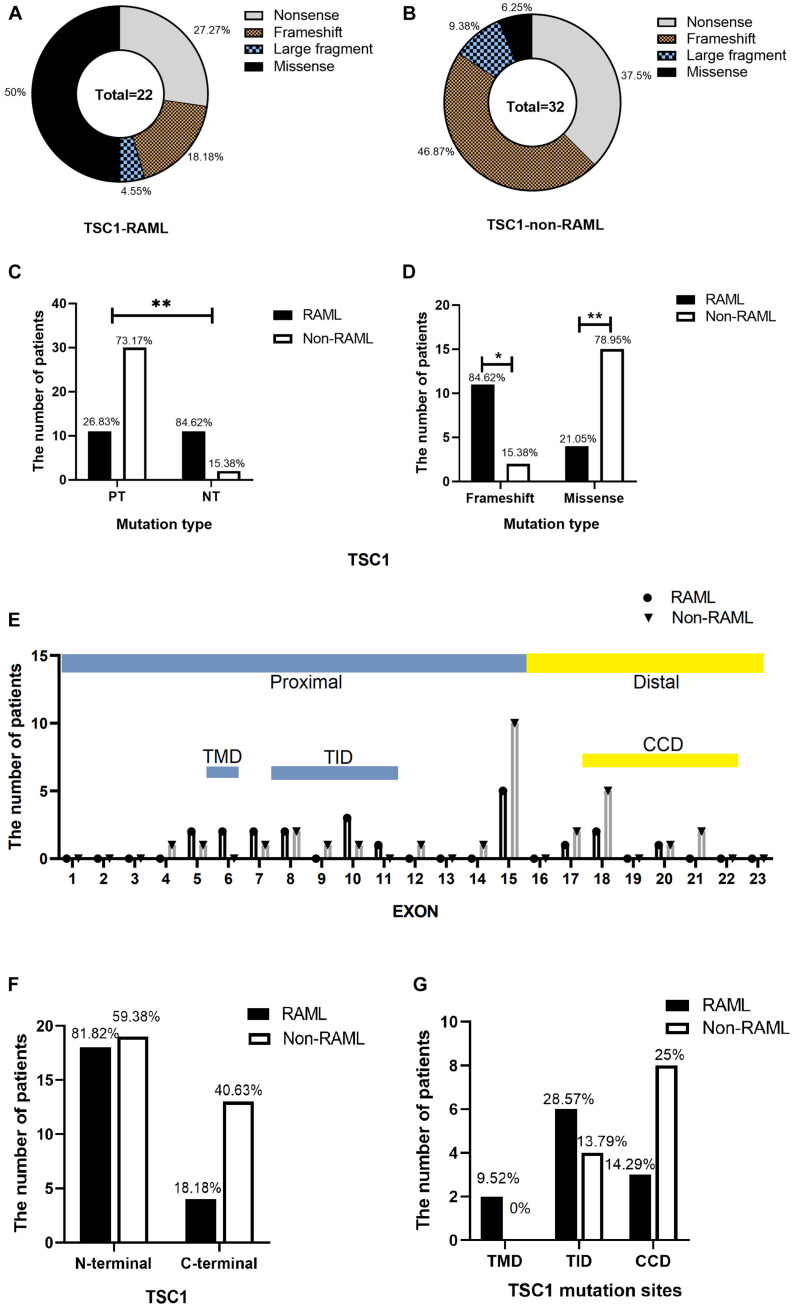
Distribution of *TSC1* gene mutations. **(A)** Categorization of *TSC1* gene mutation in patients with RAML. **(B)** *TSC1* gene mutation types without RAML. **(C)** Distribution of *TSC1* protein truncation mutation (PT) and protein non-truncation mutation (NT) in RAML and non-RAML patients. **(D)** Distribution of *TSC1* frameshift mutation and missense mutation in RAML and non-RAML patients. ^∗^*p* < 0.05, ^∗∗^*p* < 0.01. **(E)** The distribution of *TSC1* mutation sites in patients with or without RAML. TMD, transmembrane domain is encoded by exon 6. TID, tuberin interaction domain is encoded by exon 8–11. CCD, coiled coil domain is encoded by exon 18–22. **(F)** Distribution of N-terminal and C-terminal mutations of *TSC1* in RAML and non-RAML patients. **(G)** The relationship between gene mutation affected or potentially affected TMD, TID, CCD, and RAML. Transmembrane domain (TMD) is encoded by exon 6, tuberin interaction domain (TID) is encoded by exons 8-1 and coiled coil domain (CCD) is encoded by exon 18–22.

In total, 54 patients with *TSC1* mutations were included and analyzed to obtain the results in this study. The types of mutations in *TSC1* included nonsense, frameshift, large fragment, and missense mutations. Discrete types and rates of mutations in RAML patients with the *TSC1* gene mutation were observed when compared to non-RAML patients (Figures 2A,B). There were 41 PT mutations among the *TSC1* gene mutations, and 11 (26.83%) of these patients had RAML. In addition, 13 people had NT mutations, and 11 (84.62%) of these patients had RAML (Figure 2C). Therefore, PT mutations had a lower incidence of RAML than NT mutations (*p* < 0.01, OR = 0.067, 95%CI 0.13–0.35). The frameshift mutations were lower in patients with RAML (*p* = 0.03, OR = 0.252, 95%CI 0.07–0.912), and the rate of missense mutations was higher in patients with RAML (*p* < 0.01, OR = 15, 95%CI 2.859–78.691) compared with all other mutation types, except the missense mutation (Figure 2D). There was no difference between other mutation types in the groups (Table 1). However, due to the small sample size of PT and NT *TSC1* mutations included in this study, a bias might occur in our findings.

**TABLE 1:** Relationship between different types of *TSC1* gene mutation and RAML.

Mutation type	RAML (%)	Non-RAML (%)	#P	OR	95%CI
PT	11 (50%)	30 (93.75%)	<0.01**	0.067	(0.13–0.35)
Non-sense	6 (27.27%)	12 (37.5%)	0.433	1.6	(0.492–5.207)
Frameshift	4 (18.18%)	15 (46.87%)	0.03*	0.252	(0.07–0.912)
Large fragment	1 (4.55%)	3 (9.38%)	0.891&	0.46	(0.045–4.74)
NT	11 (50%)	2 (6.25%)	<0.01**	15	(2.859–78.691)
Missense	11 (50%)	2 (6.25%)	<0.01**	15	(2.859–78.691)
Total	22 (100%)	32 (100%)	−	−	−

PT, protein truncation; NT, non-protein truncation; OR, odds ratio; #, chi-square test. & represents Continuity correction. *p < 0.05. **p < 0.01. OR, odds ratio; 95%CI, 95% confidence interval.

Moreover, there were 18 (81.82%) N-terminal mutations, two (9.52%) mutations occurred in exon 6 that encoded TMD, and six (28.57%) mutations occurred in exons 8–11 that encoded TID in patients with RAML. There were four (18.18%) C-terminal mutations in the RAML group, three (14.29%) mutations occurred in exons 18–22 that encoded CCD. Nonetheless, there were 19 (59.38%) N-terminal mutations, four (13.79%) mutations in exons 8–11 that encoded TID, and 13 (40.63%) C-terminal mutations in non-RAML group. Additionally, eight (25%) mutations occurred in exons 18–22 that encoded CCD in patients without RAML. Despite having patients with or without RAML, the *TSC1* gene mutations tend to accumulate in the N- terminal region (Figure 2E). However, neither the location of the mutation nor the TMD/TID/CCD domains showed alterations in eliciting RAML (Figures 2F,G).

### Correlation Between *TSC2* Gene Mutations and RAML

**FIGURE 3:**
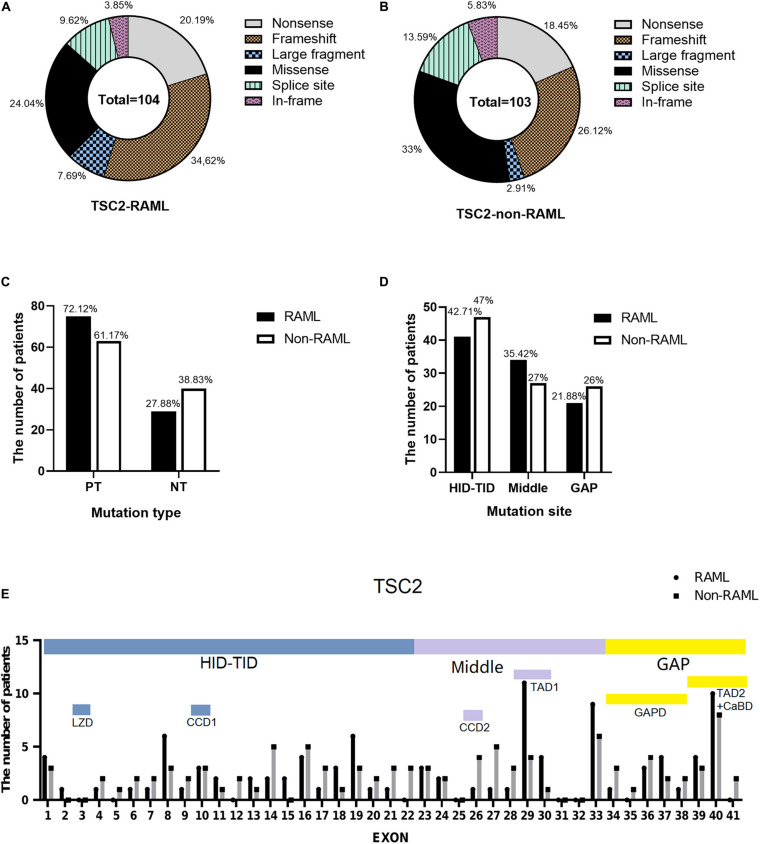
Types and distribution sites of *TSC2* gene mutations. **(A)** Mutation types of *TSC2* gene mutation in patients with RAML. **(B)** Mutation types of *TSC2* gene mutation in patients without RAML. **(C)** The relationship between *TSC2* protein truncation mutation (PT) and protein non-truncation mutation (NT) and RAML. **(D)** The relationship between gene mutation affected or potentially affected HID-TID, middle region, GAP domain and RAML. **(E)** The distribution of *TSC2* mutation sites in patients with or without RAML. HID-TID is encoded by exons 1–22, GAP domain is encoded by exons 34–41, and middle mutations between the two regions were encoded by exons 23–33. Leucine zipper domain (LZD) is encoded by exon 3, coiled coil domain 1 (CCD1) is encoded by exon 10, coiled coil domain 2 (CCD2) is encoded by exon 26, transcription activation domain 1 (TAD1) is encoded by exons 29–30, GTPase activating protein domain (GAPD) is encoded by exons 34–38, transcription activation domain 2 (TAD2) is encoded by exons 39–40 and calmodulin-binding domain (CaBD) is encoded by exons 40–41.

Among the 207 patients with *TSC2* gene mutations, 104 individuals had RAML. Additionally, 75 (72.12%) had PT mutations, which included 36 frameshift mutations (34.62%), 21 nonsense mutations (20.19%), eight large fragment deletions (7.69%) and 10 splice site mutations (9.62%). Twenty-nine had NT mutations (27.88%), which included four in-frame mutations (3.85%) and 25 missense mutations (24.04%) (Figure 3A). In contrast, 63 out of 103 non-RAML patients had PT mutations (61.17%), including 27 frameshift mutations (26.12%), 19 nonsense mutations (18.45%), 14 splice site mutations (13.59%), and three large fragment mutations (2.91%), [with two large deletion fragments and one large duplication fragment (1.95%)]. There were 40 NT mutations (38.83%) in the non-RAML subjects, which included six in-frame mutations (5.83%) and 34 missense mutations (33%) (Figure 3B). The distribution of the *TSC2* gene mutation was uniform in patients with or without RAML. In addition, the incidence of PT mutations was higher in patients with RAML when compared to those without RAML, albeit no statistical significance was observed (Figure 3C).

In the RAML patients, there were 41 (42.71%) gene mutations in the HID-TID domain and 21 (21.88%) gene mutations in exons encoding the GAP domain. There are 34 (35.42%) mutations in the middle exons of 23–33. In patients without RAML, 47 mutations (47%) were observed in the HID-TID domain, while it was 26 (26%) mutations in the GAP domain and 27 (27%) mutations in the middle coding region. Of note, *TSC2* gene mutations favored accumulation in the HID-TID coding region. Nonetheless, the location of gene mutations in the exons that encode distinct proteins showed no correlation with the occurrence of RAML disease (Figure 3D).

Interestingly, there were 41 gene mutations in exons that encoded the seven functional proteins in the *TSC2* mutation patients with RAML, which included three in CCD1, one in CCD2, 15 in TAD1, eight in GAPD, and 14 in TAD2/CaBD. On the other hand, 36 mutations were found in the same loci in patients without RAML. Briefly, there are three in CCD1, four in CCD2, five in TAD1, 11 in GAPD, and 13 in TAD2/CaBD (Figure 3E and Table 2).

**TABLE 2:** Relationship between mutations of exon gene in *TSC2* coding functional regions and RAML.

Regions	RAML N (%) *N* = 96	Non-RAML N (%) *N* = 100	#P	#OR	#95%CI	##P	Crude OR (95%CI)
LZD	0	0	−	−	−	−	−
CCD1	3 (3.13%)	3 (3%)	1&	1.043	(0.205–5.299)	0.842	−
CCD2	1 (1.04%)	4 (4%)	0.39&	0.253	(0.028–2.302)	0.236	−
TAD1	15 (15.63%)	5 (5%)	0.014*	3.518	(1.226–10.101)	0.019*	3.519 (1.226–10.101)
GAPD	8 (8.33%)	11 (11%)	0.528	0.736	(0.282–1.916)	0.717	−
TAD2 + CaBD	14 (14.58%)	13 (13%)	0.748	1.143	(0.507–2.576)	0.509	−
Others	55 (57.29%)	64 (64%)	0.336	0.755	(0.425–1.341)	0.94	−

& represents Continuity correction. #: chi-square test. ##: Binary logistic regression test, *p < 0.05, statistically significant. OR, Odds ratio; 95%CI, 95% Confidence interval. Crude OR: exp(B) of binary logistic regression test. LZD, Leucine zipper domain; CCD, coiled coil domain; TAD, transcription activation domain; GAPD, GTPase activating protein domain; CaBD, calmodulin-binding domain.

Intrigued by the results in Table 2, we inferred that the mutations that occurred in the gene encoding TAD1 led to a higher risk of RAML than any other functional regions of tuberin. Our statistical results show that the TAD1 mutation in TSC2 patients has a higher risk of RAML [*p* = 0.019, OR = 3.519 (95%CI 1.226–10.101)]. There were eight nonsense mutations, one splice site mutation, three frameshift mutations, and three missense mutations in the exons encoding TAD1 in RAML patients. On the contrary, one missense mutation, two nonsense mutations, one splice site mutation, and one frameshift mutation were observed in the same loci in patients without RAML. However, there was no statistically significant difference with regard to the distribution of mutations between the two groups.

## Discussion

Currently, there are fragmentary studies that focus on the correlation between TSC gene mutations and phenotypes. Although the genotype-phenotype correlation in patients with TSC-RAML has been conducted in many studies (Li et al., 2018; Ni et al., 2019), the sample size of these remained too small. Thus, a further analysis with a larger sample size is required. Our research included a larger study cohort by extensively searching the studies published in the last two decades and patients from multiple countries were included to make our study more convincible.

Several studies have indicated that *TSC1* mutations are associated with milder clinical outcomes in patients with TSC (Jones et al., 1999; Dabora et al., 2001; Sancak et al., 2005). In addition, the incidence of RAML is higher, and the disease is more severe in patients with *TSC2* mutations than in those with *TSC1* mutations (Rakowski et al., 2006; Au et al., 2007). However, another study revealed that only milder developmental delay and/or intellectual disability was observed in patients with *TSC1* mutations than in those with *TSC2* (Niida et al., 2013). We herein revealed that the prevalence of the *TSC2* gene mutation with RAML is higher than that of *TSC1*, without reaching a statistical difference. Moreover, female patients had a higher risk of RAML than male patients [*p* = 0.008, OR = 2.474, 95%CI (1.258–4.864)], which is in agreement with that elucidated in the previous studies (Rakowski et al., 2006; Au et al., 2007).

Regardless of the existence of RAML, mutations in the *TSC1* gene involved the trend of N- accumulation. Patients with RAML bear more frequent variants in the TID domain of the TSC1 protein versus patients without RAML, even though the difference was not statistically significant. TSC1 maintains stability, activity, and transportation of the TSC1-TSC2 complex (Rosner et al., 2008). Amino acids that are close to the N-terminal region of TSC1 are dominant in TSC1 functions. The alterations in the amino acid sequence of this region disrupt the formation of large TSC1 integration and promote proteasome-mediated TSC1 degradation, leading to an mTOR signaling cascade. Indeed, rapid degradation of the mutant TSC1 isoforms *in vivo* removed the suppressive effects of TSC1-TSC2 -variants on mTOR signaling (Mozaffari et al., 2009), promoting the progression of TSC. Among the mutations in *TSC1*, missense mutation was shown to be higher in patients with RAML [*p* < 0.05, OR = 15, 95%CI (2.859–78.691)]. Meanwhile, due to fewer amino acid substitutions, missense mutations ameliorated clinical symptoms in patients with TSC (van Eeghen et al., 2012, 2013). How the *TSC1* missense mutation contributed to the phenotype remains unclear (Nellist et al., 2009). Of interest, some missense mutations not only change at just an amino acid, but also affect the splice site. For example, the c.737G > A (p. Arg246Lys) transition is predicted to be the most likely pathogenic splice-site mutation. It was predicted to destroy the splice donor site at the 3′ end of exon 8 (Nellist et al., 2009). Specific missense mutations might lead to increased incidence of RAML in our study. However, due to the small sample size of the *TSC1* mutation included in this study, a bias might occur in our findings.

Research shows that tuberin plays a novel role in redox process which may contribute to the development of kidney tumors in patients with a tuberous sclerosis complex (Habib and Abboud, 2016). PT mutation was the most dominant category of *TSC2* mutations and was not related to the presence of RAML – this is in agreement with previous findings (Lewis et al., 2004). Uniform *TSC2* gene mutation sites are mainly located in the proximal N-terminal HID-TID functional region, in both RAML and non-RAML patients. The N-terminal region of tuberin contributed to the interaction of hamartin and tuberin (Hodges et al., 2001). Mutations that occur near the N-terminal region that potentially disturb the HID-TID functional region might hamper the formation of the TSC complex, leading to TSC progression. In addition, the GAP region of tuberin contains RapGAP homology that negatively regulates Rheb. RAML and non-RAML patients also had similar mutation rates for the genes encoding GAP. Furthermore, TAD1 is a transcriptional activation domain that is encoded by exons 29–30 (amino acids 1163–1259). Patients with mutations in the TAD1 of tuberin had a higher risk of developing RAML than patients with mutations in other regions. [*p* = 0.019, OR = 3.519, 95%CI (1.226–10.101)], albeit comparable mutation types in the TAD1 region in patients with and without RAML. The mutation in the C-terminal gene regulates the function of Rheb. The identification of TADs in TSC2 warrants an in-depth study with regard to the transcription-related function of TSC2. Indeed, TSC2 can modulate the transcriptional activity of the steroid receptor superfamily *via* binding to the estrogen receptor alpha (ERα) by TADs (Henry et al., 1998). Anzick reported the direct correlation of tumors, including breast and ovarian cancer, with the overexpression of AIB1, a member of the steroid receptor coregulators (Anzick et al., 1997). It might be hypothesized that TSC2 binding to Erα can also regulate the cell cycle and differentiation, leading to the formation of multiple system hamartomas, including RAML. TSC2 also negatively regulates the transcription level of EREG through promoter sub-regions of EREG (Pradhan et al., 2014). The upregulation of EREG has been found in several cancers and TSC patients. Mutations of TADs may disrupt the transcription factor function of TSC2 and may cause the upregulation of EREG and the occurrence of RAML. Nonetheless, these studies mainly focused on the TADs of the C-terminal region. Thus, our understanding on TAD1 function is limited and warrants further investigation.

The present study found that the missense mutation of TSC1 and the mutation of TSC2 encoding TAD1 showed a correlation with the high risk of RAML. The frameshift mutation of TSC1 also lowered the risk of RAML. This helps in early detection and intervention of TSC patients with RAML. However, PT mutations in the proximal terminal region of the gene scan leads to a base change of the distal region. Due to insufficient gender and age information of the included subjects, this study failed to correct the confounding factors, resulting in a bias. A larger study cohort with comprehensive basic information is therefore warranted to obtain more accurate results.

## Data Availability Statement

The original contributions presented in the study are included in the article/Supplementary Material, further inquiries can be directed to the corresponding author/s.

## Author Contributions

QG and HX designed the study. NZ, XW, and HX wrote the manuscript. ZT, XQ, ZG, and DH analyzed the data. All authors aided in data interpretation, reviewed, and approved the final manuscript.

## Conflict of Interest

The authors declare that the research was conducted in the absence of any commercial or financial relationships that could be construed as a potential conflict of interest.
